# Correlation between ICDAS and histology: Differences between stereomicroscopy and microradiography with contrast solution as histological techniques

**DOI:** 10.1371/journal.pone.0183432

**Published:** 2017-08-25

**Authors:** Samara de Azevedo Gomes Campos, Maria Lúcia Oliveira Vieira, Frederico Barbosa de Sousa

**Affiliations:** 1 Master program in Dentistry, Centro de Ciências da Saúde, Universidade Federal da Paraiba, Cidade Universitária, João Pessoa, Paraiba, Brasil; 2 Laboratory of Microscopy and Biological Image, Centro de Ciências da Saúde, Universidade Federal da Paraiba, Cidade Universitária, João Pessoa, Paraiba, Brasil; 3 Department of Morphology, Centro de Ciências da Saúde, Universidade Federal da Paraiba, Cidade Universitária, João Pessoa, Paraiba, Brasil; University of Washington, UNITED STATES

## Abstract

Detection of occlusal caries with visual examination using ICDAS correlates strongly with histology under stereomicroscopy (SM), but dentin aspects under SM are ambiguous regarding mineral content. Thus, our aim was to test two null hypotheses: SM and microradiography result in similar correlations between ICDAS and histology; SM and microradiography result in similar positive (PPV) and negative predictive values (NPV) of ICDAS cut-off 1–2 (scores 0–2 as sound) with histological threshold D3 (demineralization in the inner third of dentin). Occlusal surfaces of extracted permanent teeth (n = 115) were scored using ICDAS. Undemineralized ground sections were histologically scored using both SM without contrast solution and microradiography after immersion in Thoulet’s solution 1.47 for 24 h (MRC). Correlation between ICDAS and histology differed from SM (0.782) to MRC (0.511) (p = 0.0002), with a large effect size “q” of 0.49 (95% CI: 0.638/0.338). For ICDAS cut-off 1–2 and D3, PPV from MRC (0.56) was higher than that from SM (0.28) (p< 0.00001; effect size h = 0.81), and NPV from MRC (0.72) was lower than that from SM (1,00) (p < 0.00001; effect size h = 1.58). In conclusion, SM overestimated the correlation between ICDAS and lesion depth, and underestimated the number of occlusal surfaces with ICDAS cut-off 1–2 and deep dentin demineralization.

## Introduction

**Fig 1:**
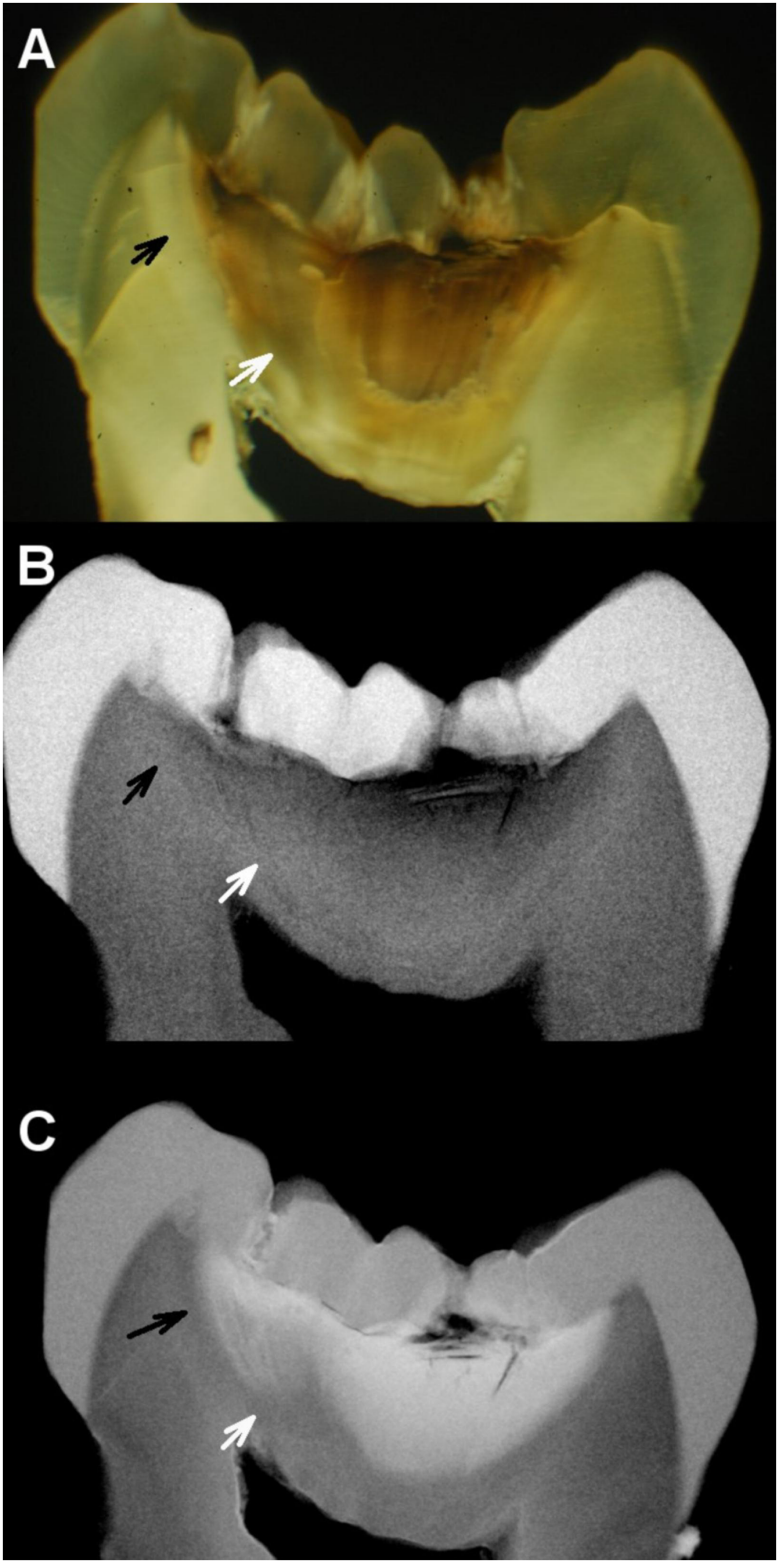
Histology of ICDAS score 4. Internal histological features of an occlusal surface with ICDAS score 4 visualized under SM (A), microradiography without contrast (B), and MRC (C), with similar lesion depth in all techniques. The contrast solution infiltrated demineralized dentin following the paths of the dentinal tubules. Both sites pointed by the black and white arrows have the same aspect (dentin sclerosis) under both microradiography without contrast (B) and MRC (C), but under SM those sites have different aspects: the site pointed by the black arrow has white opaque aspect, and the site pointed by the white arrow has a translucent aspect (A).

Visual caries detection of the occlusal tooth surface with visual scoring systems that include early noncavitated caries lesions, like the International Caries Detection and Assessment System (ICDAS), has been satisfactorily validated with the histological depth of carious demineralization measured with the aid of stereomicroscopy (SM) [1–3]. From the correlation coefficients, the higher the ICDAS score the deeper the lesion depth. The dentin layer accounts for most of the distance between the enamel occlusal surface and the roof of the coronary dental pulp chamber, so that the dentin aspects under SM are the main ones for the histological identification of deep carious lesions with SM. The aspects of translucent dentin and dentin without yellowish/brownish discolorations under SM are not included in the depth of dentin demineralization measured with SM [1–2]. It has been shown, however, that translucent dentin can be either carious or sclerotic dentin [4–9], and that dentin translucency under SM is poorly correlated with dentin radiopacity under microradiography [9] (see also Fig 1 of Bjorndal, Darvann & Thylstrup, 1988) [10]. The microradiographic identification of radiolucent dentin at the front of an occlusal caries lesion where translucent dentin is detected by SM would result in deeper dentin demineralization measured from microradiography compared to the depth of dentin demineralization measured from SM.

Microradiography is considered as a gold-standard technique for detecting variations in mineral content in hard dental tissues [9,11]. Aside from the thickness of dentin, the aspects of dentin under microradiography depend on the linear absorption coefficients (LAC) of X-rays of the main chemical components of dentin (hydroxyapatite, collagen, and water) [12]. Neglecting the LAC of water, the LAC of hydroxyapatite is one order of magnitude higher than the LAC of collagen, so that, within dentin, a decrease in radiopacity from mineral loss is not compensated by an increase in radiopacity from gain in collagen content [9,13] resulting that radiolucent dentin is not ambiguously related to low mineral content. The light scattered from dentin and detected with SM is affected by the density [14–15] and angulation of dentinal tubules [14], and the density of dentinal tubules increases form the enamel-dentin junction to the pulp [16]. Differences in refractive indexes also affect light scattering, and the refractive indexes of dentin mineral (1.62) and collagen (1.40) [15] are close enough to allow a compensation between mineral loss and collagen gain. Those factors might contribute to a misinterpretation of dentin demineralization by SM.

In this context, differences in lesion depth measured by SM and microradiography and the implication in the validation of ICDAS should be investigated. In order to identify dentin demineralization with improved contrast, microradiography with iodide solution as a contrast solution (MRC) was used, following its use to identify larger pores in rocks [17]. The aim of this study was to test two null hypotheses: (i) the correlation between the visual aspect of occlusal caries in permanent teeth detected using ICDAS and lesion depth determined with SM. is similar to the same correlation when lesion depth is determined using microradiography with contrast (MRC); and (ii) the number of both sound and caries occlusal surfaces diagnosed with ICDAS cut-off 1–2 (0–2 as sound) presenting deep dentin demineralization (histological threshold D3: demineralization in the middle third of dentin) is similar when lesion depth is measured by both SM and MRC.

## Materials and methods

### Ethical approval

This study was approved by the ethical committee on research in humans of the Lauro Wanderley Hospital of the Federal University of Paraiba in João Pessoa, Brazil (register number 39014214.0.0000.5188).

### Samples

The sample consisted of extracted human premolars and permanent third molars donated by volunteers (living in an area without water fluoridation) that provided signed free informed written consent. Teeth presenting crown fracture, calculus deposits, endodontic preparation, and dental restorations were excluded. All teeth were cleaned with pumice and rotating brush, washed with distilled water and kept in 0.02% sodium azyde solution.

### Sample size calculation

Sample size calculation for testing the difference between correlation coefficients requires predicted values of each correlation coefficient. The predicted correlation coefficient between ICDAS and lesion depth from SM was the mean of the five values [r_mean_ = (0.58+0.68+0.54+0.43+0.54)/5 = 0.554)] reported by others [3], who used the same histological scoring system [2] as the one used here. We predicted that the correlation between ICDAS and lesion depth from microradiography would not be lower than 0.25. We expected a large difference between the two correlations. The effect size for this type of hypothesis testing is Cohen’s effect size q [18]:

q=z1−z2 (1)

$$z=\frac{1}{2}\text{log}e\left(\frac{1+r}{1-r}\right)$$ (2)

Where z1 and z2 are the Fisher’s z transforms of the correlation coefficients r1 (ICDAS x SM) and r2 (ICDAS x microradiography), respectively. The effect size q of 0.369 is related to the difference between r1 of 0.544 and r2 of 0.25. Considering a one-tailed (because the difference is unidirectional only) type I error of 5% and a statistical power of 80%, a sample size of 94 was calculated with equation 4.4.1 of Cohen^18^ [3+(1240–3)/100q^2^ = 94). With an estimated loss of 22%, the final sample size was 115 teeth.

The second null hypotheses tested involved the difference between two proportions, and a large effect size was predicted. Using an effect size h of 0.8 (the smallest value for a large effect size for the difference between proportions) [18]. two-tailed alpha error of 5%, and statistical power of 80%, the sample size if 25 (Table 6.3.1 of Cohen) [18]. Thus, the sample size of 115 is adequate for testing the two null hypotheses.

### Visual examination with ICDAS

Occlusal surfaces were positioned at 25 cm from the eyes of each of two examiners, illuminated with room illumination, and examined wet and then after drying with compressed air for 5 s independently by the two examiners, who were previously trained with the e-learning program provided by the ICDAS Foundation and with 60 sample extracted teeth. The examiners applied independently the ICDAS scoring system [19] with no attempt to detect lesion activity: 0 = sound tooth surface; 1 = enamel opacity/discoloration visible after air drying; 2 = enamel opacity discoloration visible when wet, and still visible after air drying; 3 = localized breakdown in opaque/discolored enamel before and after air drying; 4 = underlying dark shadow from dentin with or without enamel breakdown; 5 = distinct dentin cavity; and 6 = distinct dentin cavity involving more than half of the occlusal surface. Intra and inter-examiners reliabilities were tested using a sample of 69 occlusal surfaces that were examined twice with a 15 days’ interval. Once calibration was achieved, a site on each occlusal surface of all teeth was selected and its location annotated in relation to the morphology of the occlusal surface. The final ICDAS score was determined by consensus between the calibrated examiners.

### Digital microradiography prior to histological sectioning

In order to select the site with the deepest lesion depth for longitudinal histological sectioning (mesio-distally to the tooth crown), each tooth was examined with real-time digital microradiography (PCBA Inspector, GE, Germany; Tungsten anode with Beryllium window, operating at 40 kV and 0.10 mA). The occlusal surface was positioned perpendicular to the X rays path, and the tooth was rotated to 360° so that a video file was produced. Each video was analyzed, the site of the deepest lesion depth was identified and its distance from the buccal surface measured. This procedure guided the histological sectioning of each occlusal surface.

### Histological examination

A 1 mm-thick longitudinal undemineralized section oriented mesio-distally to the tooth crown, cut with a diamond disc (0.15 mm thick) mounted in a low speed motor (with water irrigation), was prepared for each occlusal surface including the site where the ICDAS score was determined, as oriented by previous microradiographic examination. Each section received a consensus score of lesion depth according the following histological scoring system [2]: 0 = no demineralization in enamel; 1 = demineralization limited to the outer 50% of the enamel layer; 2 = demineralization only between the inner 50% of the enamel layer and the outer third of the dentin; 3 demineralization involving the middle third of the dentin; and 4 = demineralization involving the inner third of the dentin.

Each section was scored histologically with regard to lesion depth using both SM (without contrast solution and after drying at 25°C and 50% of air humidity for 2 h, and using 7X magnification and reflected light) and digital microradiography after immersion in contrast solution (MRC) for 24 h. A digital photograph of the histological aspect in each technique was obtained. The intra and inter-examiners reliability of the same two examiners were tested for each technique by analyzing 30 sections twice between an interval of 2 weeks. The final SM and MRC lesion depth scores were determined by consensus between examiners.

The contrast solution consisted of a Thoulet’s solution (aqueous solution of potassium iodide and mercuric iodide) with refractive index 1.47 and pH 6.7, as determined in an Abbe refractometer and in a pHmeter. This solution was used because it has been previously shown to infiltrate natural enamel caries after 24 h of immersion at room temperature [20]. The higher absorption coefficients of the components of the contrast solution result in increased opacity in the infiltrated sites.

### Data analysis

The intra and inter-examiner reliabilities on ICDAS, SM, and MRC were determined using Kappa statistics. The Spearman correlations between ICDAS and lesion depth (from SM and MRC) were calculated along with corresponding p values, 95% confidence intervals, and statistical power using the equations available elsewhere [18].

The effect size q (difference between correlations) was calculated with Eqs (1) and (2). Statistical significance was evaluated with a Z test (two-tailed alpha level of 5%) [18]. The 95% confidence interval of the effect size q was calculated using the method of Zou (2007) [21] for overlapping correlations with r-to-z Fisher’s transformations, and the statistical power was calculated using the equation 12.4.1 of Cohen [18].

We also tested the null hypothesis that the positive predictive value (true histological carious cases divided by the total number of histological carious cases) and the negative predictive value (true sound cases divided by the total of sound cases) using ICDAS cut-off 1–2 (scores 0,1, and 2 considered as sound; and scores 3, 4, 5 and 6 considered as carious) with histological threshold D3 from both SM and MRC are similar. The statistical significance was tested with a Z test (two-tailed alpha error of 5%), and the effect size h (for the difference between proportions) and the statistical power were calculated using the methods described elsewhere [18].

## Results

Intra and inter-examiner reliabilities were the following: for ICDAS, the intra-examiner Kappa scores were 0.97 and 0.97, and the inter-examiner Kappa score was 0.85; for SM, the intra-examiner Kappa scores were 0.82 and 0.89, and the inter-examiner Kappa score was 0.95; and for MRC, the intra-examiner Kappa scores were 0.94 and 0.91, and the inter-examiner Kappa score was 0.93.

There was no sample loss in this study. The distribution of ICDAS score in relation to the histological scores from both SM and MRC are shown in Table 1. The sample comprised 25 surfaces with ICDAS score 0, 39 with ICDAS score 1–2, 37 with ICDAS score 3–4, and 14 with ICDAS score 5–6 (Table 1). Forty-one occlusal surfaces with ICDAS score 0–3 presented histological score 3–4 (12 of them with ICDAS 0–1) as determined from MRC, while 27 surfaces presented that same combination of visual and histological scores (none of them with ICDAS score 0–1) when lesion depth was determined with SM. The number of occlusal surfaces with ICDAS score 0 and histological score 0 decreased from 13 (using SM) to 4 (using MRC). Most of the cases with similar lesion depths from both SM and MRC were observed for occlusal surfaces with ICDAS score 1–3 and histological score 1–2.

**Table 1:** Distribution of ICDAS scores in relation to the histological scores from SM (between brackets) and MRC (between parenthesis).

histology		ICDAS						
	0	1	2	3	4	5	6	Total
0	[06] (02)	[07] (02)	[00] (00)	[00] (00)	[00] (00)	[00] (00)	[00] (00)	[13] (04)
1	[16] (12)	[12] (13)	[02] (00)	[01] (01)	[00] (00)	[00] (00)	[00] (00)	[31] (26)
2	[03] (04)	[12] (11)	[05] (01)	[23] (10)	[02] (02)	[01] (00)	[00] (00)	[46] (28)
3	[00] (01)	[00] (00)	[01] (01)	[08] (17)	[00] (01)	[04] (01)	[02] (00)	[14] (21)
4	[00] (06)	[00] (05)	[00] (06)	[01] (05)	[02] (01)	[04] (08)	[03] (05)	[10] (36)
Total	25	31	8	33	4	9	5	115

Fig 1 shows a sample with similar histological scores from both SM and MRC for an occlusal surface with ICDAS score 4. The SM image shows brown dentin in the inner third of dentin surrounded by sites with both white opaque and translucent dentins. The microradiography without contrast solution shows radiolucency (demineralization) in the area related to brown dentin and radiopacity (indicative of sclerosis) in the areas related to both white opaque dentin and translucent dentin. The MRC image shows a highly radiopaque area matching the radiolucent area shown by microradiography without contrast solution, indicative that the infiltration of the contrast solution followed the paths of dentinal tubules in carious dentin, which, in turn, was related to the site of carious enamel on the occlusal surface. Demineralized dentin was the zone more infiltrated by contrast solution, followed by normal dentin. The sclerotic dentin was the least infiltrated by the contrast solution.

**Fig 2:**
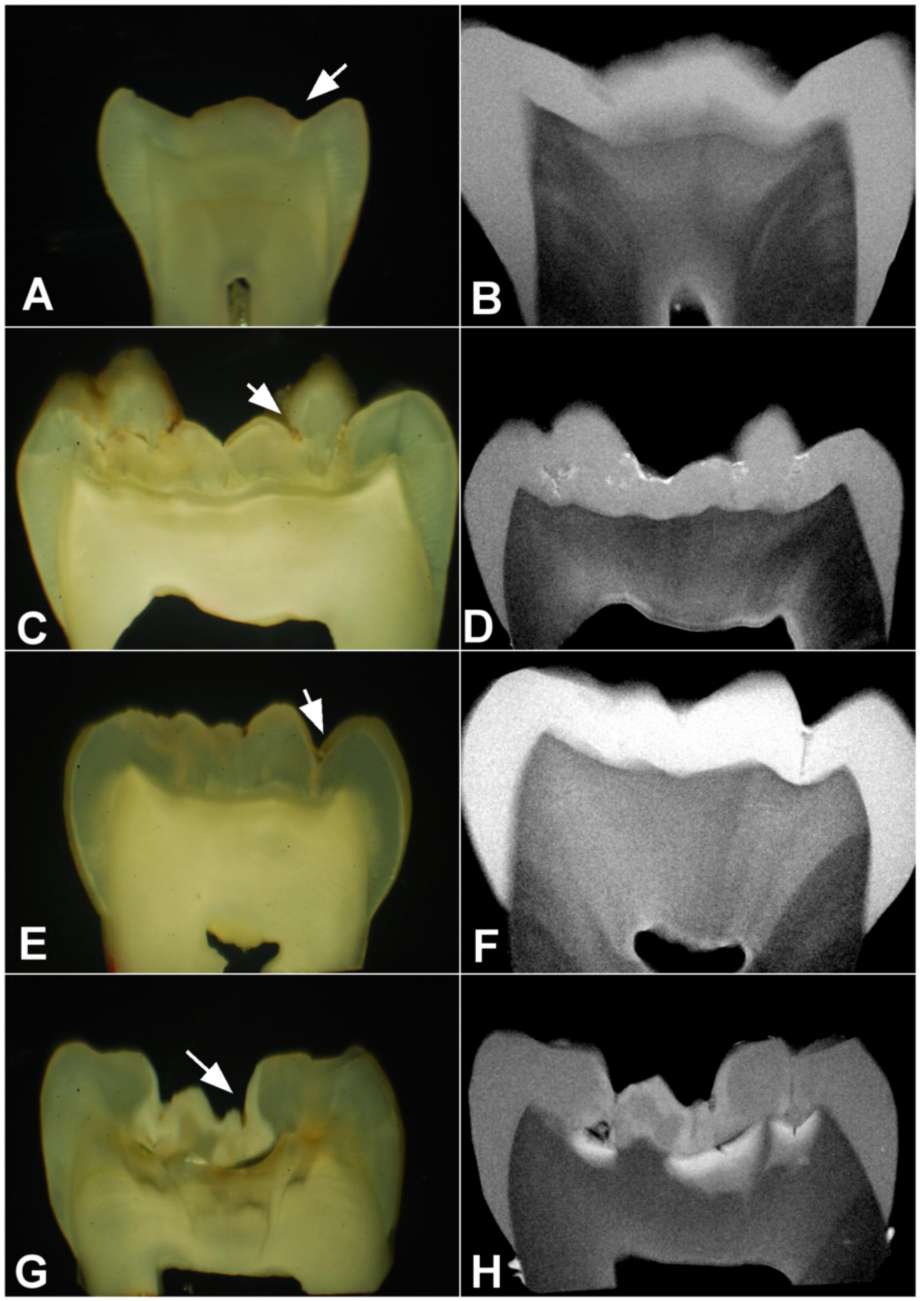
Disagreements between SM and MRC. Histological lesion depths under SM and MRC images of grounds sections of occlusal surfaces with different ICDAS scores (arrow indicates the location of ICDAS on occlusal surface): ICDAS score 0 with histological score 0 under SM (A) and histological score 4 under MRC (B); ICDAS score 1 with histological score 2 under SM (C) and histological score 4 under MRC (D); ICDAS score 2 with histological score 2 under SM (E) and histological score 4 under MRC (F); and ICDAS score 3 with histological score 2 under SM (G) and histological score 4 under MRC (H).

Fig 2 shows cases of disagreement between SM and MRC for occlusal surfaces with ICDAS scores 0, 1, 2, and 3. For all cases, caries lesion was deeper when MRC was used. For the case of ICDAS score 0, for instance, the MRC image of the ground section shows carious dentin in the inner third of dentin (and, again, following the paths if dentinal tubules related to the site of interest on the occlusal surface), while the SM image of the same ground section shows a mixture of normal and translucent dentin.

**Fig 3:**
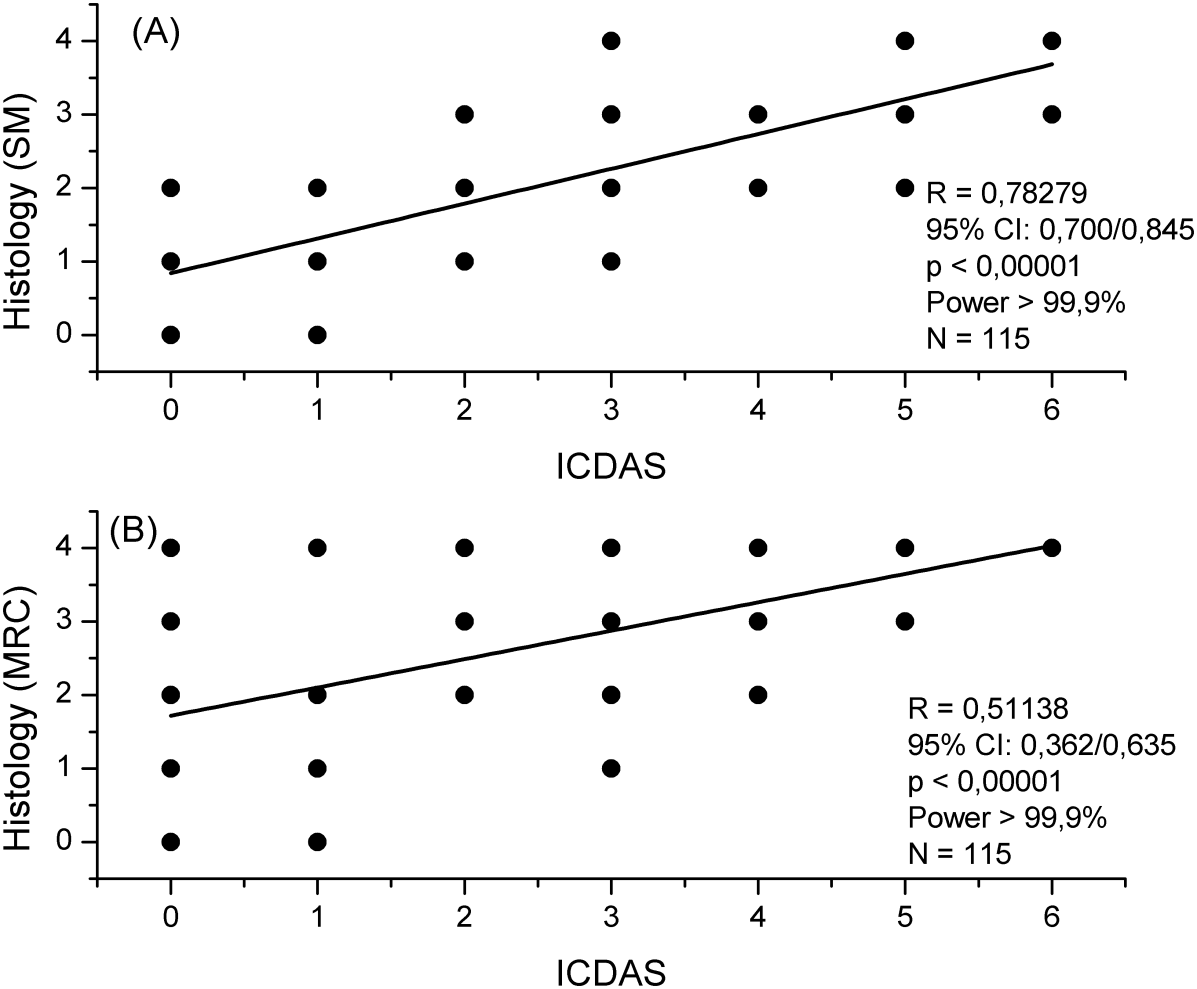
Correlation between ICDAS and histology (SM and MRC). Plots of ICDAS scores against the histological scores measured by SM (A) and MRC (B), along with corresponding correlation coefficients, 95% confidence intervals, statistical significance, and power. Most data points are overlapped; this is why there are a few filled circles.

The correlation between ICDAS and lesion depth had the correlation coefficients of 0.78289 and 0.51138, for SM and MRC, respectively (Fig 3). The difference between the two correlations was statistically significant (p < 0.0002), with and effect size q of 0.488 (95% CI = 0.638/0.338), and statistical power of 90.9%.

The PPVs for ICDAS cut-off 1–2 and histological threshold D3 (demineralization at the middle third of dentin) were the following: 0.278 using SM, and 0.555 using MRC. The corresponding NPVs were: 1.00 for SM and 0.72 for MRC. Both PPV and NPV differed statistically (p < 0.0001), with an effect size h of 0.81 for PPV and 1.58 for NPV, both with statistical power > 99.9%.

## Discussion

The correlation of 0.783 between ICDAS and lesion depth determined with SM is consistent with previous reports in the literature using similar scoring system with eight codes (0.87) [1] and five codes (0.87–0.93) [2]. It differed from the correlation between ICDAS and lesion depth determined with MRC (0.511), and the size of the difference was large (effect size q = 0.49) [18]. The difference was mainly due to increased lesion depth for ICDAS scores 0 to 3 (Table 1). We reported 22 cases of maximum lesion depth score for occlusal surfaces with ICDAS scores 0 to 3, of which six had ICDAS score 0. Occlusal surfaces with ICDAS score 0–1 and lesion depth score 3 have been previously reported when SM was used for measuring lesion depth: Jablonski-Momeni et al. [3] reported 4 cases with ICDAS score 1 and histological score D3; Diniz et al., [22] reported one case with ICDAS score 0 and histological score D3; Braga et al., [23] reported one case of ICDAS 0 and histological score D3 and 2 cases of ICDAS 1 and histological score D3. The lack of previous reports of ICDAS score 0–1 with histological score D4 might be due to factors related to the biases affecting the aspect of dentin under SM. The aspects of translucent and normal dentin under SM have been shown to be ambiguous: see our Fig 1, Fig 1 of Björndal, Darvann & Thylstrup [10], and other reports [4–9]. Those aspects do not contribute to lesion depth measured with SM. When they are confirmed as demineralization from microradiography, lesion depth increases, so that a histological score D3 from SM could increase to a histological score D4 from MRC.

In histology, carious dentin is the sum of caries-infected and caries-affected dentin, with the former including demineralized infected dentin and the later including both demineralized non-infected and sclerotic (hypermineralized) dentin [24]. Caries lesion depth in dentin is the sum of the depths of the demineralized dentin zones in both the caries-infected land caries-affected layers [1–3]. Sclerotic dentin is not included in lesion depth. Dentinal tubules are the main transport pathways in dentin [25]. Decreased peritubular dentin thickness [26] and enlargements of dentinal tubules [27] and pore sizes in intertubular dentin [28] are found in carious demineralized dentin along the path of dentinal tubules. The wider dentinal tubules and intertubular pore sizes in demineralized dentin provide more space for the penetration of the contrast solution. The potassium and mercuric iodide molecules of the contrast solution have higher X-ray absorption coefficients than the water and organic components of dentinal tubules and intertubular pores [29]. This is rational of the use of MRC for measuring lesion depth as described here.

The morphology of the dentin areas infiltrated with the contrast solution provide evidence that the infiltration of the contrast solution was not at random; it followed the demineralized dentinal tubules from the enamel-dentin junction to the roof of the dental pulp chamber, and the infiltrated dentinal tubules were related to the enamel surface of the site of choice on the occlusal surface for visual examination. The use of the Thoulet’s solution contributed to the histological examination with MRC by improving the identification of demineralized dentin (Figs 1 and 2).

Both the PPV and the NPV of ICDAS cut-off 1–2 with histological threshold D3 differed significantly from SM to MRC. The low PPV of 0.278 from SM is close to the PPV of 0.34 from SM calculated with data available in the literature [3]. The high NPV from SM of 1.0 is the same as the NPV from SM of 1.0 calculated with data available in the literature [3]. When MRC was used for the histological examination, PPV increased (PPV_MRC_ = 0.555) with a large effect size (h = 0.81), and NPV decreased (NPV_MRC_ = 0.72), also with a large effect size (h = 1.58). The PPV for ICDAS 1–2 cut-off and D3 threshold is the proportion of carious occlusal surfaces with deep dentin demineralization, while the NPV for ICDAS 1–2 cut-off and D3 threshold is the proportion of sound occlusal surfaces with deep dentin demineralization. Our results show that the proportions of both sound and carious occlusal surfaces (detected by ICDAS cut-off 1–2) with deep dentin demineralization increased significantly when lesion depth was measured with MRC. This is consistent with the recent report of occlusal caries lesion with ICDAS score 2 showing that, when lesion depth was measured histologically, SM showed only the aspect of “sound” dentin, but deep carious dentin demineralization was observed under microradiography even without contrast solution [30].

Visual scoring systems for dental caries are important for investigating the prognosis of tooth surfaces according to their different visual aspects, and thus contribute to treatment decisions. In a 4-yrs follow up study of occlusal surfaces with ICDAS score 0–4 in high-risk children, the surfaces that progressed to cavitated carious lesion at the shortest time interval included some with ICDAS scores 0 and 1 at the initial examination, while most had ICDAS score 4 at the initial examination [31]. The occurrence of occlusal surfaces with ICDAS score 0 and histological score D3 could explain the reported relatively fast transition from ICDAS score 0 to cavitated carious lesion.

In conclusion, the two null hypotheses were rejected. The correlation between ICDAS and lesion depth measured with SM was lower (with a large effect size q of 0.49) than the correlation between ICDAS and lesion depth measured with MRC. The proportions of both sound and carious occlusal surfaces diagnosed with ICDAS 1–2 cut-off presenting deep dentin demineralization detected with MRC increased significantly (with a large effect sizes; effect size h of 0.81 for PPV, and effect size h of 1.58 for NPV) compared to the same proportions when SM was used to detect dentin demineralization. This has important repercussions for the prognosis of occlusal surfaces screened by ICDAS.

## Supporting information

S1 File Data on sample code, ICDAS score, MRC score, and SM score for each occlusal surface. This is an Excel file containing tabulated data on sample code and ICDAS, MRC, and SM scores.
(XLSX)
