# Correction: Correlation between ICDAS and histology: Differences between stereomicroscopy and microradiography with contrast solution as histological techniques

**DOI:** 10.1371/journal.pone.0192270

**Published:** 2018-01-29

**Authors:** Samara de Azevedo Gomes Campos, Maria Lúcia Oliveira Vieira, Frederico Barbosa de Sousa

There is an error in the second sentence of the Abstract. The correct sentence is: Thus, our aim was to test two null hypotheses: SM and microradiography result in similar correlations between ICDAS and histology; SM and microradiography result in similar positive (PPV) and negative predictive values (NPV) of ICDAS cut-off 1–2 (scores 0–2 as sound) with histological threshold D4 (demineralization in the inner third of dentin).

There are errors in the penultimate sentence of the Abstract. The correct sentence is: For ICDAS cut-off 1–2 and D4, PPV from MRC (0.373) was higher than that from SM (0.196) (p< 0.0002; effect size h = 0.56), and NPV from MRC (0.73) was lower than that from SM (1,00) (p < 0.00001; effect size h = 1.55).

There are errors in the last sentence of the Introduction. The correct sentence is: The aim of this study was to test two null hypotheses: (i) the correlation between the visual aspect of occlusal caries in permanent teeth detected using ICDAS and lesion depth determined with SM. is similar to the same correlation when lesion depth is determined using microradiography with contrast (MRC); and (ii) the number of both sound and caries occlusal surfaces diagnosed with ICDAS cut-off 1–2 (0–2 as sound) presenting deep dentin demineralization (histological threshold D4: demineralization in the inner third of dentin) is similar when lesion depth is measured by both SM and MRC.

The first sentence of the third paragraph beneath the “Data analysis” heading of the Materials and Methods section is incorrect. The correct sentence is: We also tested the null hypothesis that the positive predictive value (PPV = true carious cases with histological score D4 divided by the total number of carious cases with ICDAS scores 3–6) and the negative predictive value (NPV = true sound cases with histological scores 0–3 divided by the total of sound cases with ICDAS scores 0–2) using ICDAS cut-off 1–2 (scores 0,1, and 2 considered as sound; and scores 3, 4, 5 and 6 considered as carious) with histological threshold D4 from both SM and MRC are similar.

The final and penultimate sentences of the Results section are incorrect. The correct sentences are: The PPVs for sound occlusal surfaces (ICDAS scores 0, 1, and 2) and histological threshold D4 (demineralization at the inner third of dentin) were the following: 0.196 using SM, and 0.373 using MRC. The corresponding NPVs were: 1.00 for SM and 0.73 for MRC. Both PPV and NPV differed statistically (p < 0.0002), with an effect size h of 0.56 for PPV and 1.55 for NPV, both with statistical power > 96.0%.

The final sentence of the second paragraph of the Discussion section contains a typographical error. The correct sentence is: This is the rational of the use of MRC for measuring lesion depth as described here.

The first sentence of the fourth paragraph of the Discussion section contains errors. The correct sentence is: Both the PPV and the NPV of ICDAS cut-off 1–2 with histological threshold D4 differed significantly from SM to MRC. The low PPV of 0.196 from SM is close to the PPV of 0.162 from SM calculated with data available in the literature [3].

The third and fourth sentences of the fourth paragraph of the Discussion section contain errors. The correct sentences are: When MRC was used for the histological examination, PPV increased (PPV_MRC_ = 0.373) with a medium effect size (h = 0.56), and NPV decreased (NPV_MRC_ = 0.73), also with a large effect size (h = 1.55). The PPV for ICDAS 1–2 cut-off and D4 threshold is the proportion of carious occlusal surfaces with deep dentin demineralization, while the NPV for ICDAS 1–2 cut-off and D4 threshold is the proportion of sound occlusal surfaces with deep dentin demineralization.

The final sentence of the penultimate paragraph of the Discussion section contains an error. The correct sentence is: The occurrence of occlusal surfaces with ICDAS score 0 and histological score D4 could explain the reported relatively fast transition from ICDAS score 0 to cavitated carious lesion.

The first sentence of the final paragraph of the Discussion section is incorrect. The correct sentence is: In conclusion, the two null hypotheses were rejected. The correlation between ICDAS and lesion depth measured with SM was higher (with a large effect size q of 0.49) than the correlation between ICDAS and lesion depth measured with MRC.

The penultimate sentence of the final paragraph of the Discussion section contains errors. The correct sentence is: The proportions of both sound and carious occlusal surfaces diagnosed with ICDAS 1–2 cut-off presenting deep dentin demineralization detected with MRC increased significantly (with a medium effect size h of 0.56 for PPV, and a large effect size h of 1.55 for NPV) compared to the same proportions when SM was used to detect dentin demineralization.
